# Automated tracking of morphologic changes in weekly magnetic resonance imaging during head and neck radiotherapy

**DOI:** 10.1002/acm2.13959

**Published:** 2023-05-05

**Authors:** Eric Aliotta, Yu‐Chi Hu, Peng Zhang, Phillip Lichtenwalner, Amanda Caringi, Natasha Allgood, C. Jillian Tsai, Kaveh Zakeri, Nancy Lee, Pengpeng Zhang, Laura Cerviño, Michalis Aristophanous

**Affiliations:** ^1^ Department of Medical Physics Memorial Sloan Kettering Cancer Center New York New York USA; ^2^ Department of Radiation Oncology Memorial Sloan Kettering Cancer Center New York New York USA

**Keywords:** adaptive radiotherapy, head and neck, MRI‐guidance, treatment response

## Abstract

**Background and Purpose:**

Anatomic changes during head and neck radiotherapy can impact dose delivery, necessitate adaptive replanning, and indicate patient‐specific response to treatment. We have developed an automated system to track these changes through longitudinal MRI scans to aid identification and clinical intervention. The purpose of this article is to describe this tracking system and present results from an initial cohort of patients.

**Materials and Methods:**

The Automated Watchdog in Adaptive Radiotherapy Environment (AWARE) was developed to process longitudinal MRI data for radiotherapy patients. AWARE automatically identifies and collects weekly scans, propagates radiotherapy planning structures, computes structure changes over time, and reports important trends to the clinical team. AWARE also incorporates manual structure review and revision from clinical experts and dynamically updates tracking statistics when necessary. AWARE was applied to patients receiving weekly T2‐weighted MRI scans during head and neck radiotherapy. Changes in nodal gross tumor volume (GTV) and parotid gland delineations were tracked over time to assess changes during treatment and identify early indicators of treatment response.

**Results:**

*N* = 91 patients were tracked and analyzed in this study. Nodal GTVs and parotids both shrunk considerably throughout treatment (−9.7 ± 7.7% and −3.7 ± 3.3% per week, respectively). Ipsilateral parotids shrunk significantly faster than contralateral (−4.3 ± 3.1% vs. −2.9 ± 3.3% per week, *p* = 0.005) and increased in distance from GTVs over time (+2.7 ± 7.2% per week, *p* < 1 × 10^−5^). Automatic structure propagations agreed well with manual revisions (Dice = 0.88 ± 0.09 for parotids and 0.80 ± 0.15 for GTVs), but for GTVs the agreement degraded 4–5 weeks after the start of treatment. Changes in GTV volume observed by AWARE as early as one week into treatment were predictive of large changes later in the course (AUC = 0.79).

**Conclusion:**

AWARE automatically identified longitudinal changes in GTV and parotid volumes during radiotherapy. Results suggest that this system may be useful for identifying rapidly responding patients as early as one week into treatment.

## 1 INTRODUCTION

Radiation therapy is an important treatment for head and neck cancer that can non‐operatively control disease and improve survival.^1^ One notable characteristic of head and neck radiotherapy is that significant morphologic changes may occur over the course of treatment. Progressive changes due to weight loss, tumor regression, and parotid gland shrinkage are well documented during radiotherapy^2^, ^3^, ^4^, ^5^ and can have important clinical consequences. For example, because treatment plans are optimized based on pre‐treatment anatomy, changes in anatomy lead to systematic deviations between planned and delivered dose distributions that can reduce target coverage and increase doses to normal tissue^6^, ^7^, ^8^, ^9^ unless addressed with an adaptive plan modification.^3^, ^7^, ^10^, ^11^, ^12^ Changes in tumor morphology may also have prognostic value in that they provide patient‐specific indicators of treatment response, which may be predictive of outcomes.^13^, ^14^, ^15^, ^16^ The ability to identify and track anatomic changes in real‐time during radiotherapy would therefore have substantial clinical value.

One way to assess anatomic changes during radiotherapy is to perform longitudinal imaging at multiple timepoints and observe any changes to relevant structures. MRI is an ideal technique for this purpose due to its excellent visualization of head and neck anatomy^17^, ^18^ and lack of ionizing radiation. Furthermore, MRI is increasingly available for routine imaging in radiotherapy clinics as MR‐Linacs^19^, ^20^ and MR‐Simulators^21^, ^22^ become widespread. However, even with this data available, extracting relevant morphologic changes from a series of MRIs requires substantial image processing and analysis that can be time‐consuming and labor‐intensive. For this information to be clinically actionable, longitudinal trends must be identified and reported in real‐time. An automated approach is clearly necessary for clinical implementation at scale.

In this study, we describe an automated system that was developed to accommodate high‐throughput and real‐time analysis of longitudinal MRI scans for patients undergoing radiotherapy. This system automatically detects and deformably co‐registers weekly scans for active patients, propagates radiotherapy planning structures, tracks changes in structure morphology, and reports relevant trends to the clinical team. The purpose of this article is to describe the implementation of this system in our clinic and report on tracking data collected in an initial cohort of head and neck radiotherapy patients.

## 2 METHODS

### 2.1 Automated MRI monitoring

**FIGURE 1:**
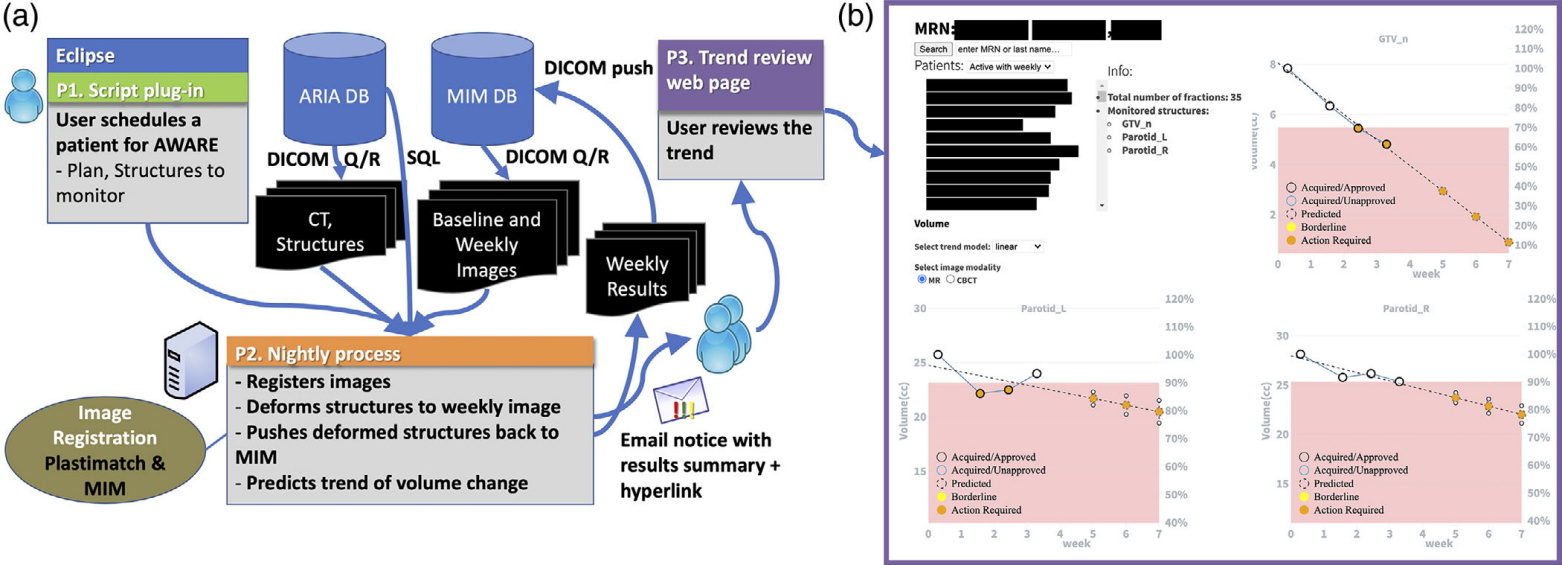
Flowchart (a) depicting the processes underlying the AWARE system including. The system begins with a user‐initiated enrollment process in an Eclipse script which is followed by nightly queries/retrieves (Q/R) from the MIM database (DB) and registrations to the planning scan. Processed results are communicated with the clinical team via automated emails and a simple web interface. Volume trend data as seen through the web interface is shown for an example patient (b). In the web interface, all tracked patients are visible in a searchable list. All tracked structures are displayed for the selected patient. Each data point displays the absolute and relative volumes as well as the approval status (i.e., whether or not it was reviewed/revised by a member of the team). Point color indicates the degree of change relative to clinical thresholds for review and potential adaptation (30% change for GTVs and 10% for parotids). “Predicted” structure volumes are generated based on a linear fit to the existing data. AWARE, Automated Watchdog in Adaptive Radiotherapy Environment.

An in‐house program, named the Automated Watchdog in Adaptive Radiotherapy Environment (AWARE),^23^ was developed to automatically identify, collect, and process longitudinal imaging data. This system, which was written in the C# programming language, involves several steps and interacts with multiple clinical databases. As such, all data were maintained within our institutional firewall at every stage in the process. A flowchart depicting the AWARE system is shown in Figure 1 and the following paragraphs detail the specifics of each component process.

#### 2.1.1 Patient identification and enrollment

First, patients who are scheduled to receive repeated MRIs during the course of treatment are identified using automated queries of our hospital scheduling system. The clinical team is then notified that these patients will be receiving longitudinal MRIs and prompted to enroll them for tracking using a custom “scheduler” script in the Eclipse treatment planning system (Varian Medical Systems, Palo Alto, CA). The scheduler script accesses the patient's treatment plan information from ARIA via the Eclipse application programming interface (API) and prompts the user to select specific structures to be monitored throughout treatment. Information about the selected structures (structure IDs and baseline volumes), along with plan information such as prescription dose, fractionation, and dose volume histogram (DVH) statistics are then exported to an Access database (Microsoft, Redmond, Washington). Dicom unique identifiers (UIDs) for the planning CT and structure set are also exported to facilitate automatic data retrieval later in the process.

#### 2.1.2 Image retrieval

Once a patient is enrolled in AWARE tracking, the system performs a nightly search for new scans in PACs matching their medical record number (MRN) and collects any newly available scans using DICOM query/retrieve commands. The system looks for T2 weighted scans that were acquired as part of our standard head and neck imaging protocol (identified via standardized series descriptions). Once identified, AWARE collects these images and imports them into our MIM database (MIM software, Beachwood, OH).

#### 2.1.3 Structure propagation

When a new scan is identified, AWARE automatically performs deformable registration to map the baseline planning structures to the latest MRI. An initial deformable registration between the planning CT and initial MRI is performed using a multimodality mutual‐information algorithm implemented in Plastimatch (plastimatch.org). MRI‐to‐MRI registration is then performed to map the pre‐treatment MRI to the latest MRI using the built‐in deformable registration algorithm in MIM (accessed through customized automated workflows). All registrations are performed automatically with no user input and were optimized to minimize differences across the entire patient anatomy. This deformable mapping is then used to propagate all tracking structures to the latest MRI. Individual structure volumes are then computed and pushed to the Access database and the propagated structure set is saved to the MIM database.

#### 2.1.4 Communication with clinical team

Following contour propagation, an automated email is sent to the clinical team to indicate that new data is available and provide a report of current volumetric trends. Data and trends are also viewable through a web page that pulls from the AWARE database (Figure 1). A member of the clinical team (either a physicist or dosimetrist) then reviews the latest scan and structures in MIM, makes any necessary adjustments to propagated structures to maintain consistency with the original MD‐approved delineations, and saves a new structure set to MIM. AWARE will then identify these updated structures in the following nightly process and update volumetric trends with the reviewed structure volumes.

### 2.2 Patient cohort

AWARE was used to track patients undergoing chemoradiation therapy for head and neck cancer and receiving weekly MRI examinations during treatment as part of their clinical care. Because longitudinal MRI is not standard of care for head and neck radiotherapy, these were generally patients being treated on a clinical trial which required additional imaging.^24^ The collection and analysis of this data was approved by the local institutional review board.

All patients were treated on conventional linear accelerators (Varian C‐series or TrueBeam) using either intensity modulated radiation therapy (IMRT) or volumetric modulated arc therapy (VMAT) with CT‐based treatment planning. Nodal gross tumor volumes (GTV) and parotids glands were selected as tracking structures for all patients. Because the majority of the patients in this cohort had their primary tumors resected prior to radiotherapy, primary GTVs were not included in this study.

Weekly MRI was performed on a Philips 3T MR Simulator (Koninklijke Philips, Amsterdam, Netherlands) using a T2‐weighted 2D‐multislice turbo‐spin‐echo sequence with fat suppression applied via Spectral Attenuated Inversion Recovery (SPAIR). This fat‐suppressed T2‐weighted protocol was used because it provides excellent soft‐tissue contrast in the head and neck area, particularly in identifying involved lymph nodes from surrounding tissue, without the need for an exogenous contrast agent. All scans were acquired with the patient in the head‐first supine position. Pre‐treatment scans were acquired with the patient in the treatment position with a thermoplastic immobilization mask in place, while on‐treatment scans were acquired in a standard radiological position and without the immobilization mask in place.

### 2.3 Data analysis

Patients with longitudinal MRIs identified by AWARE for at least three timepoints were included for analysis. At each timepoint, the volume of each nodal GTV and parotid structure was normalized to its baseline volume (from the initial MRI) to generate trends of relative volume over time. Because scans were not acquired at fixed timepoints (although they were acquired once per week, in general), the trends were linearly interpolated to even one‐week increments at 7, 14, 21, 28, and 35 days after the start of RT and low‐pass filtered using the “smooth” function in MATLAB (The Mathworks, Natick, MA). These trends were used to determine the average weekly volume changes for GTVs and parotids across all patients. Per‐week volume changes were computed via differences in relative volume between subsequent weeks. To determine the impact of the interpolation and smoothing post‐processing steps, we additionally compared the un‐smoothed and non‐interpolated volume change measurements using paired t‐tests.

#### 2.3.1 Assessing parotid‐GTV distance

Because the distance between parotid glands and target volumes are an important predictor of mean parotid dose (an important metric regarding treatment toxicity),^25^, ^26^, ^27^, ^28^ systematic changes in this distance may have significant implications for adaptive therapy. Furthermore, this type of analysis can easily be integrated in AWARE. Therefore, the mean distance between each parotid and GTV was assessed at each time point. This calculation, performed using custom MATLAB code, involved first computing the distance between each voxel within a parotid and its nearest GTV outer surface to produce a map of distance‐to‐GTV within each parotid gland. Regions of parotid that overlapped with GTV were assigned distances of 0. The mean distance across the full three‐dimensional volume of the parotid then described the average parotid‐to‐GTV distance.

At each MRI timepoint, the average GTV distance was computed for each parotid and trended against time as described above. The baseline parotid‐to‐GTV distance (obtained from the initial MRI) was also used to identify parotids as either ipsilateral (<5 cm) or contralateral (≥5 cm) for each plan. Note that in the case of multilateral disease, both parotids could be considered ipsilateral for a given patient.

#### 2.3.2 Comparison of deformed and manually edited structures

Although integration of the AWARE system with MIM clinical software made routine contour review and modification straightforward, the need for manual input undoubtedly reduces efficiency and limits throughput compared to a fully automated system. To assess the feasibility of using AWARE as a fully automated system, deformably propagated structures were compared with manually revised structures. Comparisons were performed using the Dice overlap coefficient and percentage volume difference (ΔV) between structures. Dice and ΔV were computed for each subject and on each weekly scan. Significant changes in dice and ΔV as a function of time (weeks) were evaluated using analysis of variance (ANOVA). At each week, the presence of significant volume biases was tested using single‐tailed t‐tests in comparison with a distribution mean of 0.

#### 2.3.3 Early predictors of major GTV changes

We then assessed whether metrics that can be identified pre‐treatment or in the first week of treatment and can be fully automated within AWARE can predict major changes later in the course. For this analysis, we divided all cases according to baseline GTV volume and the first measure of GTV volume change. To simulate the real clinical scenario, only information that would be available in real‐time was used for this analysis and the initial volume change was measured directly from the first week's scan (rather than the interpolated and smoothed trend used for population analysis).

Two methods were used to evaluate predictors of volumetric change. In the first method, patients were divided according to the median population value for each metric and trends of GTV change over time were assessed for each group. Overall volume changes were assessed between groups at each subsequent week using two‐tailed t‐tests (significance level = 0.05). While volume changes were tracked up to five weeks after the start of treatment, statistical comparisons were restricted to weeks 1−3 because not all patients received radiation at weeks 4 and 5. Bonferroni corrections were applied to limit type‐1 error by multiplying *p*‐values by the number of comparisons made.

For the second method, rather than dividing cases based on the population median, an optimal threshold value was chosen to maximize the accuracy of predicting large GTV volume changes at week 3. In this method, patients were grouped into small versus large total changes if their relative week 3 GTV volume was greater than versus less than the population median (at week 3). Receiver operator curve (ROC) analysis was then used to identify the threshold initial GTV change which optimally predicted this grouping. As in the statistical analysis, week 3 was chosen as the timepoint for assessing major changes because it was the latest timepoint at which all patients in the cohort were still receiving radiation.

This analysis was then repeated using deformed (non‐edited) structures to assess the predictive value of a fully automated pipeline. In this analysis, deformed week 1 structures were used to predict week 2−3 volume changes as measured by the manually reviewed/revised structures (i.e., the reviewed structures were always used as the prediction endpoint). As described above, differences in week 2−3 volumetric change were assessed based on deformed GTV changes observed at week 1. ROC analysis was also repeated to identify an optimal cutoff for predicting week 3 volume changes based on the week 1 deformed volume.

## 3 RESULTS

A total of 132 patients were enrolled for tracking with AWARE. Of these, 22 were excluded from this study due to a lack of weekly manual contour review, and 19 were excluded due to either a lack of a baseline scan within the first 5 days of treatment or fewer than three longitudinal scans. This left a total of *N* = 91 patients for analysis. This cohort included a total of 106 tracked nodal GTVs (some patients had multiple GTVs) and 176 parotid volumes (three patients were missing parotid tracking data) that were available for assessment.

Most of these patients had human papilloma virus (HPV) positive cancers (*N* = 88), many of which (*N* = 67) were treated to a low‐dose 30 Gy radiotherapy protocol delivered in 15 fractions (2 Gy per fraction).^29^ The other 24 patients were treated to a total dose of 70 Gy in two phases: a 15‐fraction initial phase followed by a 20‐fraction boost (both phases at 2 Gy per fraction). Additional patient information is shown in Table 1. Note that while 30 Gy patients only received treatment for 3 weeks, they still received weekly MRI in weeks 4 and 5 (though no radiation was being delivered in these weeks). The results that follow all include patients from both groups.

**TABLE 1:** Patient demographics for the 91 patients included in analysis.

Age	
Median	59
Range	41–80
Gender	
Male	84 (92%)
Female	7 (8%)
HPV status	
Positive	88 (97%)
Negative	2 (2%)
Unknown	1 (1%)
Rx Dose	
70 Gy (2 Gy x 35 fractions)	24 (26%)
30 Gy (2 Gy x 15 fractions)	67 (74%)
Site	
Oropharynx	89 (98%)
Nasopharynx	1 (1%)
Hypopharynx	1 (1%)

### 3.1 GTV and parotid changes

**FIGURE 2:**
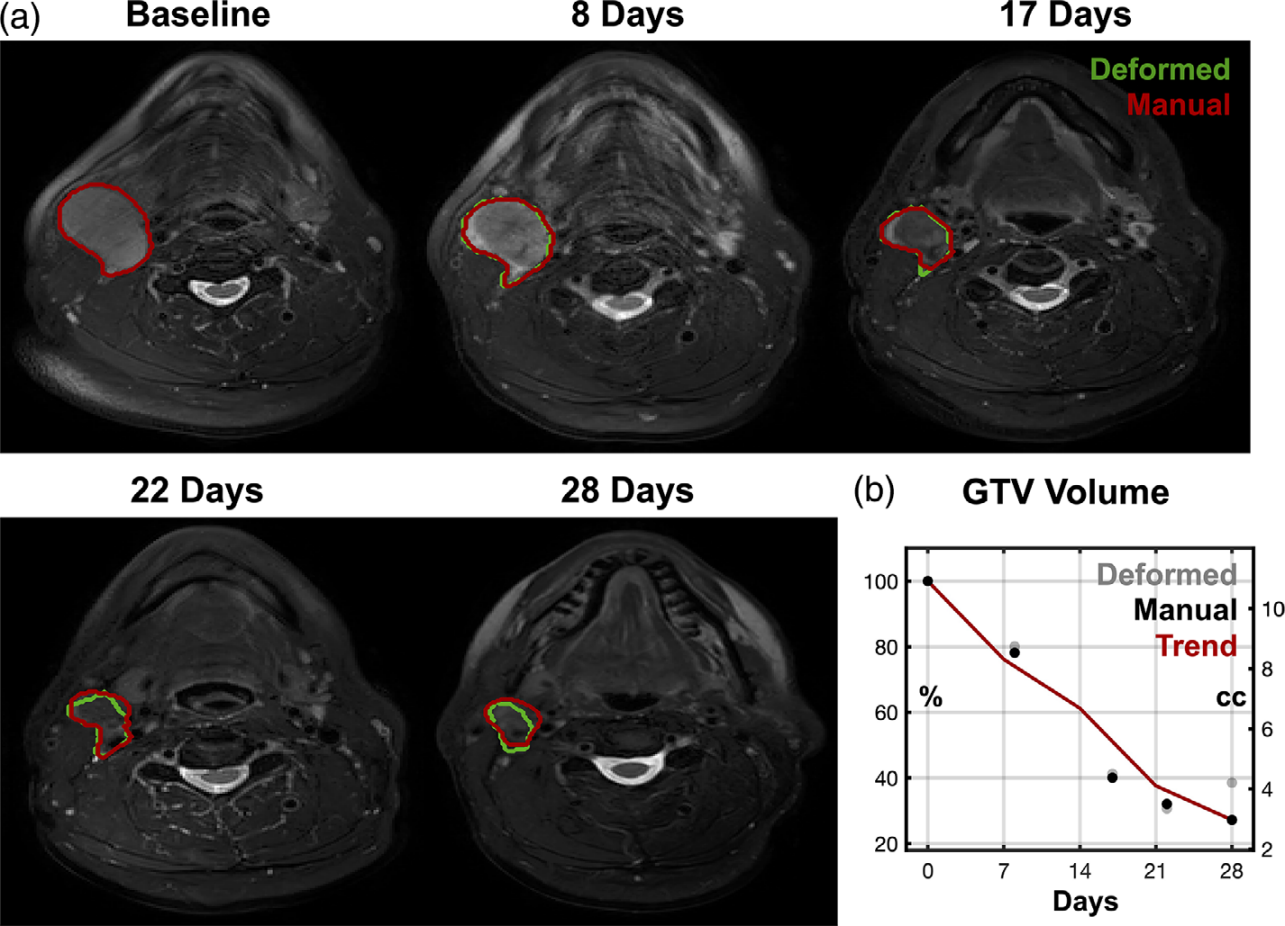
Example images and propagated nodal GTV volumes throughout a course of radiotherapy (a). Green contours indicate the deformed volumes directly output from automated image analysis and red contours indicate manually edited volumes. Over time, this GTV showed a clear trend (b) of volume shrinkage over time with >20% regression at the first weekly scan and >60% regression by end of treatment. GTV, gross tumor volume.

**FIGURE 3:**
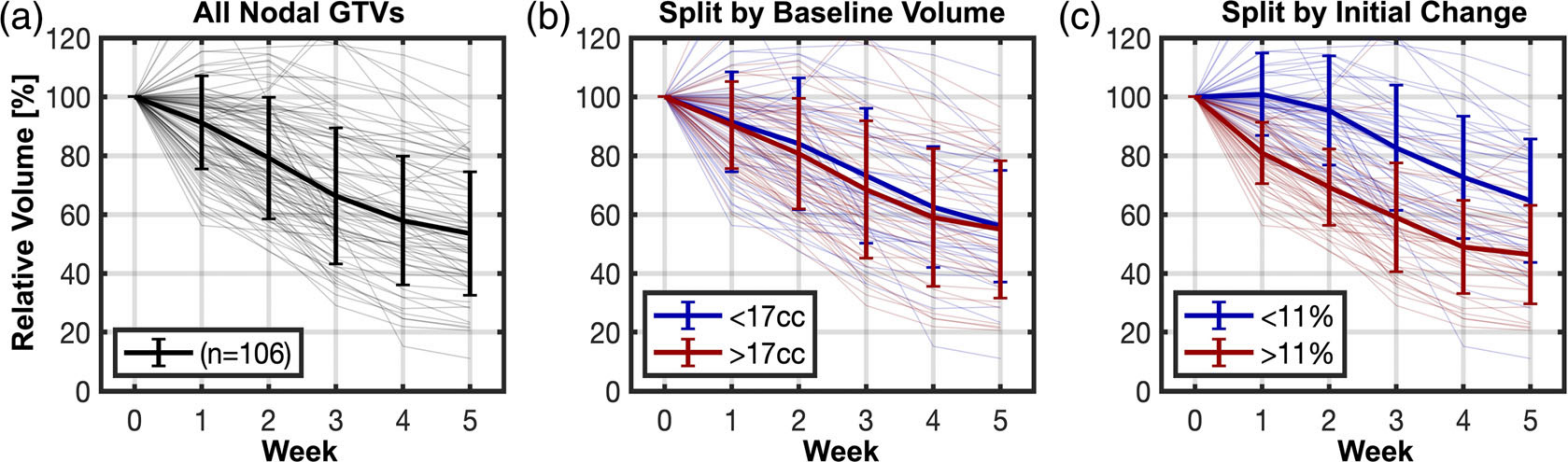
GTV volume trends during radiotherapy are plotted for the full cohort of patients (a). Thin lines show volume trends for individual patients while thick error bars show the population mean ± one standard deviation. No significant effect was observed as a function of whether a GTV's baseline volume was greater than or less than the population median (b), but significant variability was observed based on the initial measure of volume change (c). GTVs with initial regression larger than the median (red) continued to show increased regression compared to other cases at subsequent weeks (blue). Note that for patients treated to a total dose of 30 Gy, radiation was only delivered in the first 3 weeks (though volume data is shown for all weeks and patients). GTV, gross tumor volume.

An example time series of longitudinal scans and GTV delineations captured by AWARE is shown in Figure 2. GTV volume changes over time are plotted across the entire cohort in Figure 3. Across all patients, GTVs started with a baseline volume of 19.5 ± 15.4 cc (mean ± 1 standard deviation) which reduced to 13.4 ± 10.9 cc after 3 weeks of treatment. The average GTV volume reduction rate was −9.7 ± 7.7% per week, with consistent rates of change week to week (ANOVA, *p* = 0.17). There was no significant difference in GTV volume change rate with prescription dose over the 3 weeks of treatment (30 Gy: −9.8 ± 7.7% per week vs. 70 Gy: −7.8 ± 7.2% per week, *p* = 0.2). We also found that repeating the analysis without smoothing or interpolation applied to raw GTV volume data did not have a significant impact on volume change measurements (average difference: −0.34 ± 18.1%, *p* = 0.8).

**FIGURE 4:**
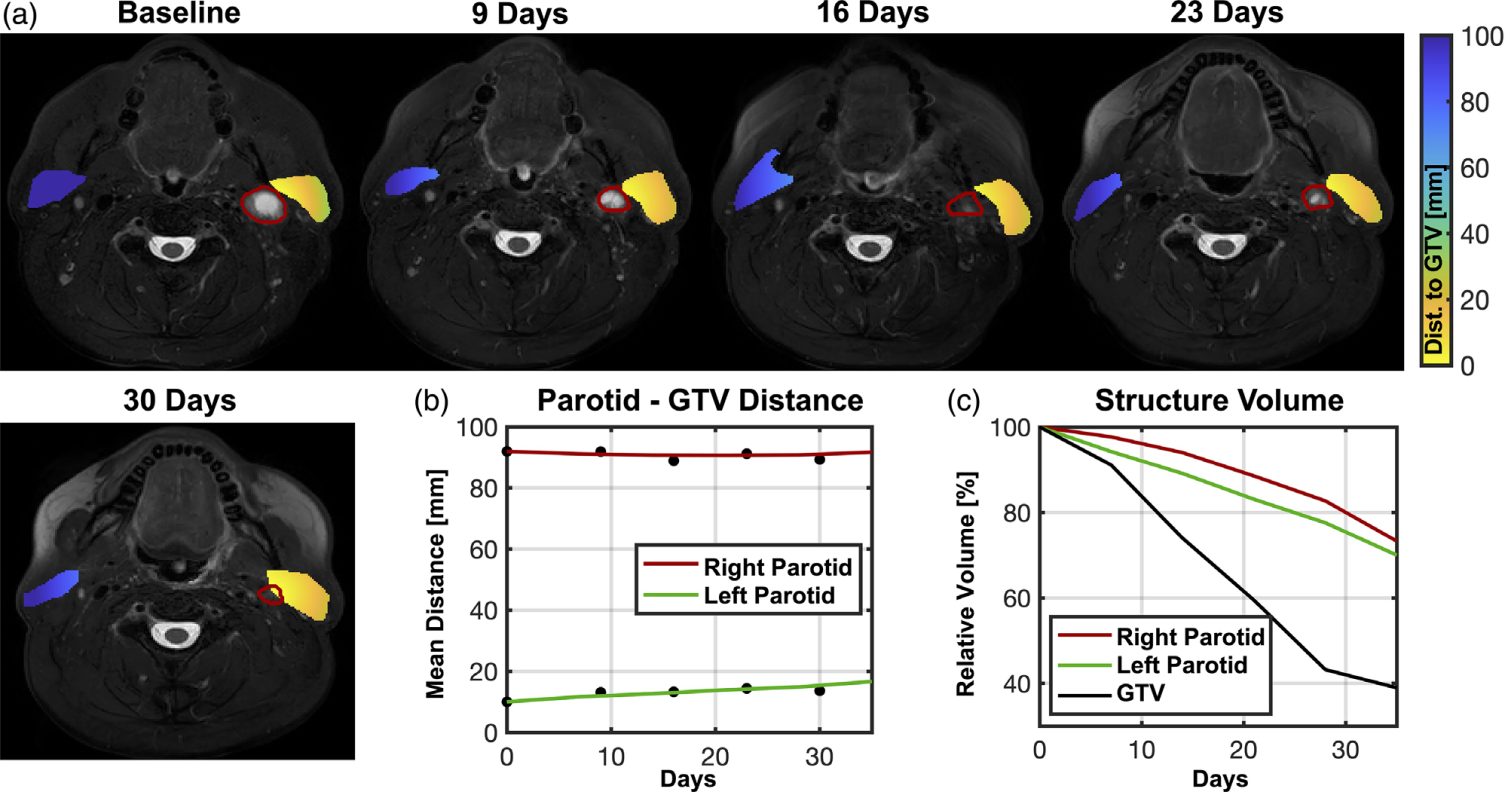
Example longitudinal images with contours propagated to each timepoint (a). Red contours indicate the nodal GTV. The overlaid colormap indicates the distance between each point within the parotid gland and the nearest surface of the GTV. Plots show the mean distance between each parotid and the GTV (averaged over all voxels) over time (b) as well as the relative volume of each structure over time (bottom right). The GTV, left, and right parotids all decreased in volume throughout treatment. The GTV shrunk faster than either parotid, and the ipsilateral (c) parotid shrunk faster than the contralateral. The left parotid increased in distance from the GTV by ∼7 mm (10 mm at baseline versus >17 mm at 30 days) while the contralateral parotid maintained a relatively constant distance. GTV, gross tumor volume.

**FIGURE 5:**
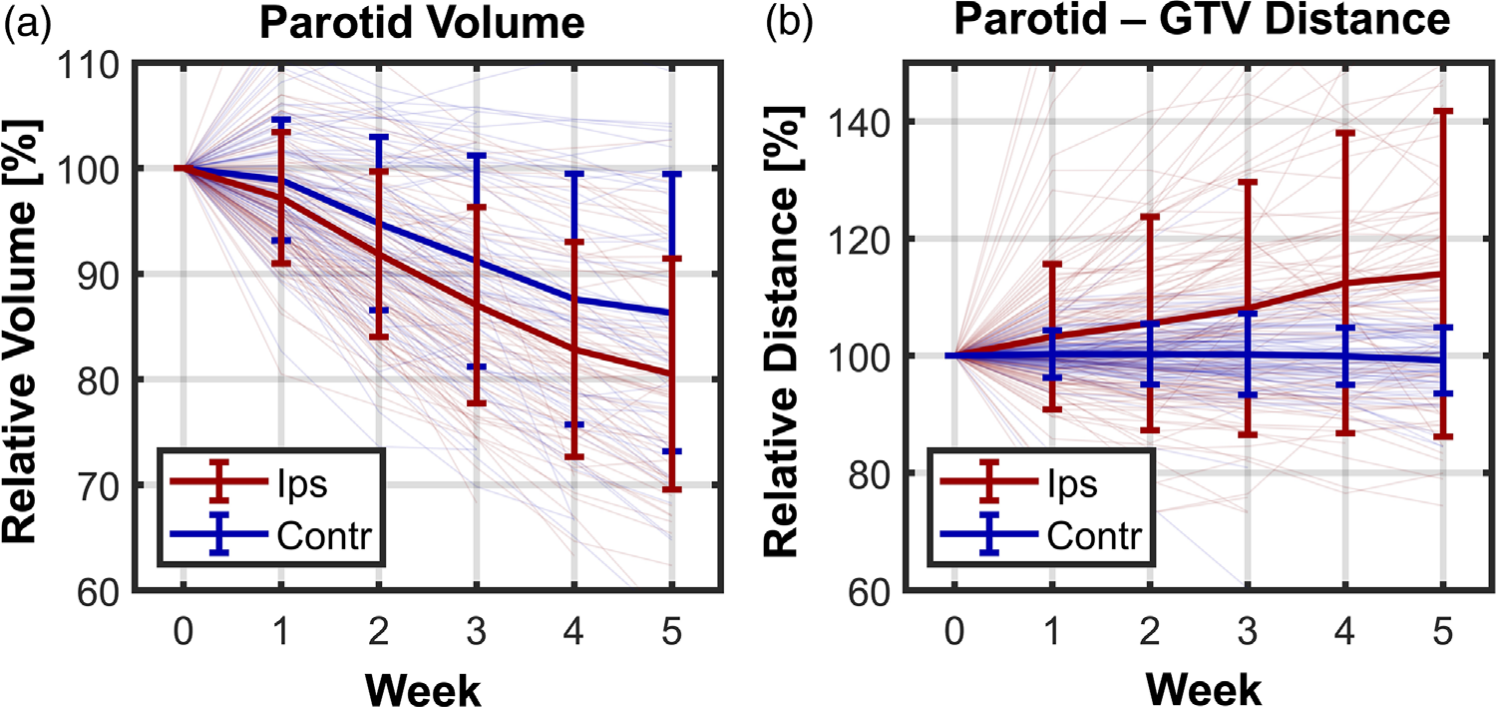
Parotid volume trends during radiotherapy plotted across all patients (a). Thin lines show trends for individual patients while thick error bars show the population mean ± one standard deviation. Parotids shrunk, in general, during treatment, and ipsilateral parotid glands (defined by parotids initially <5 cm from the GTV on average—plotted in red) shrunk at a significantly faster rate than contralateral glands (≥5 cm average distance to GTV—plotted in blue). Mean distances between ipsilateral parotid volumes and GTVs increased during treatment (b, shown in red). Distances between contralateral parotids and GTVs did not change significantly over time (blue). GTV, gross tumor volume.

An example image series showing parotid and GTV changes in one patient is shown in Figure 4 and population parotid change data are shown in Figure 5. Overall, parotids shrunk at an average rate of −3.7 ± 3.3% per week. While ipsilateral parotids shrunk −4.3 ± 3.1% per week, contralateral parotids shrunk at a significantly slower rate of −2.9 ± 3.3% per week (Figure 5, *p* = 0.005). The mean distance between parotids and GTVs increased over time by an average of +1.6 ± 5.7% per week (*p* = 2 × 10^−4^). This change was driven primarily by ipsilateral parotids (*N* = 98) which separated significantly from GTVs at a rate of +2.7 ± 7.2% per week (*p* < 1 × 10^−5^, Figure 5). Note that some patients had more than one parotid classified as ipsilateral due to proximity (<5 cm) of both parotids to a GTV. Contralateral parotids (i.e., those ≥5 cm from the GTV, *N* = 78), did not separate significantly from GTVs (+0.1 ± 2.3% per week, *p* = 0.7).

### 3.2 Comparison of deformed and manually edited structures

Agreement between automatically propagated and manually revised GTV and parotid volumes are shown in Table 2. Across all cases, deformed GTVs overlapped with manually edited volumes with an average Dice coefficient of 0.80 ± 0.15. Overlap was slightly higher for parotids (Dice = 0.88 ± 0.09). Average volume differences (ΔV) were +7.8 ± 41.3% for GTVs and −0.2 ± 13.0% for parotids. Agreement between deformed and manual structures generally became poorer with each passing week, but only the change in GTV ΔV was statistically significant. In weeks 1−3, GTV ΔV were not significantly different from 0, but GTV ΔV became significantly larger (and positive) over time (*p* = 0.02 at week 4, *p* = 0.01 at week 5) indicating that deformed structures overestimated residual GTV volumes at later weeks. Deformed parotids showed a slight, non‐significant decrease in Dice coefficient with each passing week and showed no directional volume bias at any week.

**TABLE 2:** Metrics of agreement (mean ± 1 standard deviation) between deformed and manually revised structures.

	Week 1	Week 2	Week 3	Week 4	Week 5
Dice—GTV	0.83 ± 0.15	0.82 ± 0.16	0.80 ± 0.13	0.79 ± 0.15	0.77 ± 0.16
Dice—Parotid	0.88 ± 0.13	0.89 ± 0.08	0.89 ± 0.07	0.88 ± 0.08	0.86 ± 0.06
ΔV—GTV	+0.5 ± 23.0%	+5.2 ± 36.4%	+8.5 ± 42.8%	+12.7 ± 47.2%*	+20.6 ± 60.5%*
ΔV—Parotid	−1.6 ± 15.1%	+0.2 ± 11.8%	+0.7 ± 10.8%	+1.1 ± 13.4%	−2.2 ± 15.0%

*Note*: Positive volume differences (ΔV) indicate that deformed volumes were larger. ΔV distributions that differed significantly from 0 (*p* < 0.05) and therefore indicating a significant bias are indicated by *.

### 3.3 Predictors of GTV changes

Baseline GTV volumes did not correlate significantly with rates of GTV change. GTVs with baseline volumes larger than the median (17 cc) did not shrink at a significantly faster rate than those with smaller baseline volumes (Figure 3, −10.5 ± 7.8% vs. −8.9 ± 7.6% per week, *p* = 0.3). Overall GTV volume shrinkage was not significantly different at any week for large compared with small baseline GTV volumes (all *p* > 0.2).

Initial GTV volume changes were significantly associated with later GTV regression. The first measure of GTV volume change (which was acquired 8.6 ± 3.5 days after start of treatment) showed a median volume reduction of 11%. GTVs with initial changes greater than 11% (i.e., greater shrinkage) showed significantly larger overall volume shrinkage at each subsequent week: −30.7 ± 13.0% versus −4.6 ± 18.5% at week 2 (*p* < 1 × 10^−5^) and −41.0 ± 18.5% versus −17.3 ± 21.3% at week 3 (*p* < 1 × 10^−5^). Overall shrinkages were also significantly larger for these GTVs in weeks 4 (−51.0 ± 15.8% vs. −27.4 ± 20.8%, *p* < 1 × 10^−5^) and 5 (−53.6 ± 16.7% vs. −35.3 ± 21.0%, *p* = 7 × 10^−4^), though not all patients were still undergoing treatment at those timepoints.

Week 1 GTV volume change predicted major volume changes at week 3 (i.e., greater than the week‐3 median value of −33.7%) with an area under the ROC curve (AUC) of 0.79. The optimal week 1 shrinkage cutoff for predicting large changes at week 3 was 8.3% and this cutoff achieved 74% accuracy (sensitivity/specificity = 81%/66%). Using the population median (11%) as the cutoff reduced accuracy only slightly to 72% (sensitivity/specificity = 72%/72%).

Deformed (non‐edited) GTV volume changes predicted subsequent regression with similar accuracy to manually edited volumes. The median initial volume change according to deformed GTVs was 13% and deformed GTVs with initial changes greater than this value continued to show significantly larger overall volume shrinkage at each subsequent week: −28.0 ± 13.9% versus −7.5 ± 20.6% at week 2 (*p* < 1 × 10^−5^) and −40.2 ± 18.7% versus −18.7 ± 23.2% at week 3 (*p* < 1 × 10^−5^). The AUC for predicting GTV changes at week 3 was 0.77 when deformed volumes were used and the optimal week 1 cutoff value of 11% achieved an accuracy of 73% (sensitivity/specificity = 77%/68%). Note that in these comparisons, evaluated shrinkage values were computed based on manually reviewed structures and the initial deformed structure was used only for prediction.

## 4 DISCUSSION

The AWARE system successfully automated the prospective collection and processing of longitudinal MRI data from on‐treatment head and neck radiotherapy patients in our clinic. This system permitted efficient tracking of morphologic changes during treatment and provided timely reports to the clinical team. While the current study used this data to passively monitor changes as they occurred and validate the process, ongoing efforts are using this data to intervene clinically with a targeted adaptive radiotherapy strategy.^30^ The analysis of morphological changes observed with AWARE in this initial patient cohort will also aid further development towards streamlining early interventions.

While automation is a central component of AWARE, it was specifically designed to incorporate the manual review and revision of tracking structures in a streamlined fashion. This is important for clinical operation because it is known that deformable image registration can introduce errors in structure propagation that require manual correction.^31^ The ability to easily correct these errors and have tracking statistics update automatically is a key feature for practical workflow integration. To accomplish this, AWARE interacted directly with our clinical contouring system (MIM) and performed multiple passes of the database to find updated structure sets when available and updated trends accordingly. It is worth noting that in the proposed workflow, this review and correction was performed by a physicist or dosimetrist rather than a radiation oncologist and therefore propagated GTVs are not suitable to be used directly in treatment planning. This information is only intended to identify morphologic trends which can flag a patient for adaptive replanning. The radiation oncologist then needs to be a part of the decision of whether to adapt the treatment plan. And once a decision is made to adapt, the physician would ultimately need to adjust and/or approve updated volumes.

Despite the importance of manual input and review, the comparisons between deformed and edited structures (Table 2) did show good overall agreement for GTVs (average Dice = 0.80) and Parotid glands (average Dice = 0.88). Notably, no systematic bias in volume was reported for parotids in any week or for GTVs in the first 3 weeks of treatment. This indicates that fully automated analysis may be sufficient for volume tracking in the early stages of treatment. This is further supported by the similar performance of deformed and manual week‐1 volume changes in predicting large regression at week 3 (AUC = 0.77 vs. 0.79). Future advances in deformable image registration and autosegmentation may improve contour propagation at later timepoints and further reduce the need for user intervention. However, with current capabilities, one can consider using fully automated analysis to track patients in the early weeks of treatment and flag patients with large changes for manual review in later weeks.

The data collected with AWARE demonstrated that both nodal GTVs and parotid glands shrunk consistently throughout radiotherapy at rates of −9.7 ± 7.7% and −3.7 ± 3.3% per week, respectively. These changes were consistent with other reports in the literature. For example, Barker et al. reported GTV volume changes of −12.7% per week and parotid volume changes of −4.2% per week.^3^ Similar GTV change rates have also been reported elsewhere.^32^, ^33^ Others have reported comparatively larger changes in parotid volume during radiotherapy on the order of −6% to 8% per week.^34^, ^35^ However, it has been noted that parotid shrinkage is correlated with parotid dose^34^, ^36^ and differences in planning or delivery techniques between these studies may explain the differences.

The observation that week 1 changes in nodal GTVs were predictive of significantly larger changes in subsequent weeks may aid in data‐driven patient selection for adaptive radiotherapy. Adapting radiotherapy treatment plans in response to anatomic changes is important for maintaining plan quality and reducing toxicity,^3^, ^7^, ^10^, ^11^ but time and resource constraints make it infeasible to perform on all patients. Our results indicate that GTV volume changes measured between a pre‐treatment baseline and a week 1 scan may be sufficient to select patients who will benefit greatly from adaptation. Given the strong correlations that have been observed between parotid‐to‐target distances and mean parotid doses,^25^, ^26^, ^27^, ^28^ the observed increases in distance between ipsilateral parotids and GTVs further indicate that plan adaptation may translate to reductions in toxicity for these patients. Parotid‐to‐target distance could also serve as an independent predictor for the need for adaptation.

Other groups have developed methods to inform the need for adaptive radiotherapy. Some have used pre‐treatment characteristics to predict which patients are likely to eventually need an adaptive replan.^33^, ^37^, ^38^, ^39^ Others have used changes in longitudinal cone‐beam CT (CBCT) imaging characteristics, either measured quantitatively^40^ or reported manually^41^ to identify replanning candidates. Longitudinal dose accumulation strategies have also been used to more directly assess the dosimetric impact of anatomic changes by either computing original treatment plans on updated anatomy or deformably mapping planned dose maps to an updated scan.^42^, ^43^, ^44^ AWARE could complement existing dose accumulation strategies by incorporating offline MRI information (which provides superior soft tissue discrimination in the head and neck compared with CBCT) and leveraging expert feedback on propagated structures to improve the accuracy of accumulated DVH statistics. Importantly, AWARE was developed to run prospectively and work within clinical workflows to enable tracking at‐scale and with minimal clinical overhead.

In addition to supporting adaptive radiotherapy strategies, the longitudinal tracking afforded by AWARE may have clinical value in patient‐specific response assessment. Multiple studies have found rates of target volume regression during radiotherapy to be predictive of outcomes in head and neck cancer.^13^, ^14^, ^15^, ^16^ Others have found longitudinal parotid changes to predict the onset of xerostomia after radiotherapy.^45^ With further evaluation, volume trends captured by AWARE may be useful for characterizing individual treatment response and informing personalized prognoses. In addition to changes in morphology, changes in quantitative MRI techniques such as Diffusion Weighted (DWI) and Dynamic Contrast Enhanced (DCE) imaging have shown promise in head and neck cancer outcome prediction.^46^, ^47^, ^48^, ^49^ AWARE could be extended to incorporate quantitative parameter maps and automatically track biomarkers such as the apparent diffusion coefficient (ADC) throughout treatment.

While the MRIs in this study were all “offline” scans acquired outside of treatment sessions, MR‐guided radiotherapy (MRgRT) treatment units are now available and provide excellent opportunities for “online” imaging and adaptive head and neck radiotherapy.^50^, ^51^, ^52^, ^53^ The AWARE system could be applied to patients being treated with MRgRT and automatically track changes in daily setup MRIs. This approach has a clear logistical advantage compared with offline scanning in that is does not require additional scan appointments. The automated processing of AWARE could be particularly advantageous in the context of daily MRI due to the large quantity of imaging data to be analyzed.

Online volumetric imaging is of course also routinely acquired on conventional linear accelerators equipped with CBCT. Tracking changes on CBCT images would also eliminate the need for additional imaging sessions and this is currently being assessed with the AWARE system.^30^ CBCT‐based tracking would certainly be more widely applicable than MRI as it is a standard of care technique for head and neck radiotherapy, while longitudinal MRI is not. Accurate CBCT‐based tracking of the parotid glands has been shown to be feasible,^45^ but it remains to be seen whether the lack of soft‐tissue contrast and limited field‐of‐view of CBCT impacts the ability to automatically track target volumes and/or organs‐at‐risk accurately compared to MRI. Particularly in the context of target volumes, the lack of on‐board contrast agent in CBCT may limit the ability to accurately propagate delineations.

This study does have some limitations that should be noted. While MRI is becoming more widely available within radiotherapy clinics, longitudinal MRI assessment is not currently a widespread practice, which could limit the applicability of this technique. Furthermore, while MRI is useful for visualizing head and neck anatomy, radiotherapy treatment planning currently relies on CT for dose calculation. Therefore, a decision to adapt based on MRI still generally requires that an additional CT scan be acquired for treatment planning and dose calculation (though future iterations of MR‐only simulation should eventually eliminate this limitation^54^). Another limitation was the relatively homogeneous patient cohort that was analyzed. In this study, we were limited to patients who were already undergoing weekly MRI as part of their care and this skewed heavily towards male and HPV+ patients who had their primary tumor resected and were treated on a low‐dose radiotherapy protocol.^29^ While we did not observe significantly different GTV changes amongst patients on this protocol, this population may represent a favorable cohort for radiotherapy response and the volume changes reported in this study may not be generalizable to a broader population. Furthermore, it is possible that the results observed in nodal GTVs will not generalize fully to primary tumor volumes. Finally, it is worth noting that tracking GTV and parotid volumes were reviewed and edited by a physicist or a dosimetrist rather than a radiation oncologist (though the treating radiation oncologist did define and approve the initial GTVs used in the treatment plan). While ideally, the treating physician would review all volumes (particularly GTVs), we determined this would not be feasible on a routine basis.

In conclusion, we have implemented a system for automatically tracking intra‐treatment longitudinal MRI scans in head and neck radiotherapy patients. This system permitted the efficient tracking of anatomic changes at‐scale and with minimal clinical overhead. In data collected through AWARE, we have observed changes in target and parotid volumes during treatment which may inform patient‐specific response to radiotherapy and plan adaptation strategies.

## AUTHOR CONTRIBUTIONS

*Study design, data analysis, data interpretation, and manuscript writing*: Eric Aliotta. *Study design, data analysis and collection, data interpretation, and manuscript review/approval*: Yu‐Chi Hu, Peng Zhang, Phil Lichtenwalner, Amanda Caringi, and Natasha Allgood. *Study conception and design, data interpretation, and manuscript review/approval*: Jillian Tsai, Kaveh Zakeri, and Nancy Lee. *Study conception and design, data interpretation, and manuscript review/approval*: Peng Zhang, Laura Cerviño, and Michalis Aristophanous.

## CONFLICT OF INTEREST STATEMENT

The authors declare no conflicts of interest.
